# Altered electrical properties in skeletal muscle of mice with glycogen storage disease type II

**DOI:** 10.1038/s41598-022-09328-0

**Published:** 2022-03-29

**Authors:** Janice A. Nagy, Carson Semple, Daniela Riveros, Benjamin Sanchez, Seward B. Rutkove

**Affiliations:** 1 Department of Neurology, Beth Israel Deaconess Medical Center, Harvard Medical School, Boston, MA 02215 USA; 2 Department of Electrical and Computer Engineering, University of Utah, Salt Lake City, UT 84132 USA

**Keywords:** Biophysics, Neurology

## Abstract

Electrical impedance methods, including electrical impedance myography, are increasingly being used as biomarkers of muscle health since they measure passive electrical properties of muscle that alter in disease. One disorder, Pompe Disease (Glycogen storage disease type II (GSDII)), remains relatively unstudied. This disease is marked by dramatic accumulation of intracellular myofiber glycogen. Here we assessed the electrical properties of skeletal muscle in a model of GSDII, the Pompe^*6neo*/*6neo*^ (Pompe) mouse. Ex vivo impedance measurements of gastrocnemius (GA) were obtained using a dielectric measuring cell in 30-week-old female Pompe (N = 10) and WT (N = 10) mice. Longitudinal and transverse conductivity, σ, and the relative permittivity, ε_r_, and Cole–Cole complex resistivity parameters at 0 Hz and infinite frequency, ρ_o_ and ρ_∞_, respectively, and the intracellular resistivity, ρ_intracellular_ were determined from the impedance data. Glycogen content (GC) was visualized histologically and quantified biochemically. At frequencies > 1 MHz, Pompe mice demonstrated significantly decreased longitudinal and transverse conductivity, increased Cole–Cole parameters, ρ_o_ and ρ_o_-ρ_∞_, and decreased ρ_intracellular_. Changes in longitudinal conductivity and ρ_intracellular_ correlated with increased GC in Pompe animals. Ex vivo high frequency impedance measures are sensitive to alterations in intracellular myofiber features considered characteristic of GSDII, making them potentially useful measures of disease status.

## Introduction

Quantitative techniques are needed to serve as biomarkers for the assessment of disease progression and therapeutic efficacy in a variety of disorders affecting skeletal muscle. One approach that has offered potential value for this purpose is electrical impedance myography (EIM)^1–4^. In EIM, a low-amplitude, high-frequency electrical current is passed through a muscle of interest and the resultant voltage is measured. Disease-induced alterations in muscle volume electrical conduction properties will impact the measured electrical impedance values associated with ionic and polarization alterations due to the presence of free water, connective tissue, and changes in cellular integrity. Major advantages of EIM over the other technologies include its relatively low cost, convenience, and high reproducibility^5^.

Earlier studies on isolated muscle fibers using impedance techniques provided data on the dielectric properties of skeletal muscle tissue^1,3,6^. Our own impedance measurements on excised mouse muscle have confirmed the ability of ex vivo EIM to detect disease-induced alterations in myofiber cross sectional area (CSA), ^7^ to distinguish different neuromuscular disorders^8^, and with the application of very high frequency current, to differentiate slow- versus fast-twitch muscle fibers^9^. More recently, we have focused on obtaining similar information such as myofiber CSA by using an in vivo non-invasive surface impedance approach coupled with statistical analysis to establish a relationship between impedance values and muscle fibers of varying size, including those from immature animals^10^, and in a murine neuromuscular disease model associated with prominent muscle atrophy^11^. We have also assessed changes in the *extracellular* space by the analysis of impedance data in several murine models including: inflammatory infiltrates in carrageenan-induced inflammation^12^; increased fat deposition in the db/db obese diabetic mouse^13^; and increased fibrosis in the D2-mdx model of Duchenne Muscular Dystrophy (DMD)^14^.

Here, we begin to address the effects of *intracellular* pathological abnormalities on electrical impedance by studying the effects of glycogen accumulation, using a mouse model of Pompe disease^15,16^. Pompe^*6neo*^^/*6neo*^ (also known as Pompe) mice exhibit muscular features typical of patients with Glycogen storage disease type II (GSDII), a recessively inherited deficiency of the enzyme acid α-glucosidase (GAA, acid maltase, EC 3.2.1.20). Mice homozygous for disruption of the acid α-glucosidase gene begin to accumulate glycogen in cardiac and skeletal muscle lysosomes by 3 weeks of age and develop a substantial accumulation of glycogen in both intra-lysosomal and intra-cytoplasmic compartments by 6 weeks of age. By 1 month of age, Pompe mice exhibit markedly reduced mobility and strength, and by 8–9 months of age they develop obvious muscle wasting and a weak, waddling gait. Of note, female Pompe mice are considerably more affected than males in this model^16^.

We hypothesized that intracellular myofiber abnormalities associated with Pompe disease would produce alterations in the electrical conduction properties of muscle. A total of 20 female mice (10 Pompe and 10 WT) at 30 weeks of age were assessed using ex vivo EIM measurements on the excised gastrocnemius (GA) muscle after animal euthanasia. Intracellular glycogen content (GC) in the GA muscle was visualized histologically by Periodic Acid Schiff (PAS) staining and quantified biochemically. We had two goals: 1. To evaluate differences in electrical properties between Pompe and wild-type (WT) muscle, and 2. To determine if electrical properties and/or modelled impedance parameters were associated with intramuscular GC. We hypothesized that increased GC would lead to increased intracellular water leading to reduced intracellular resistivity values and that ex vivo EIM would detect such a change.

## Results

### Physiological Measurements

**Figure 1:**
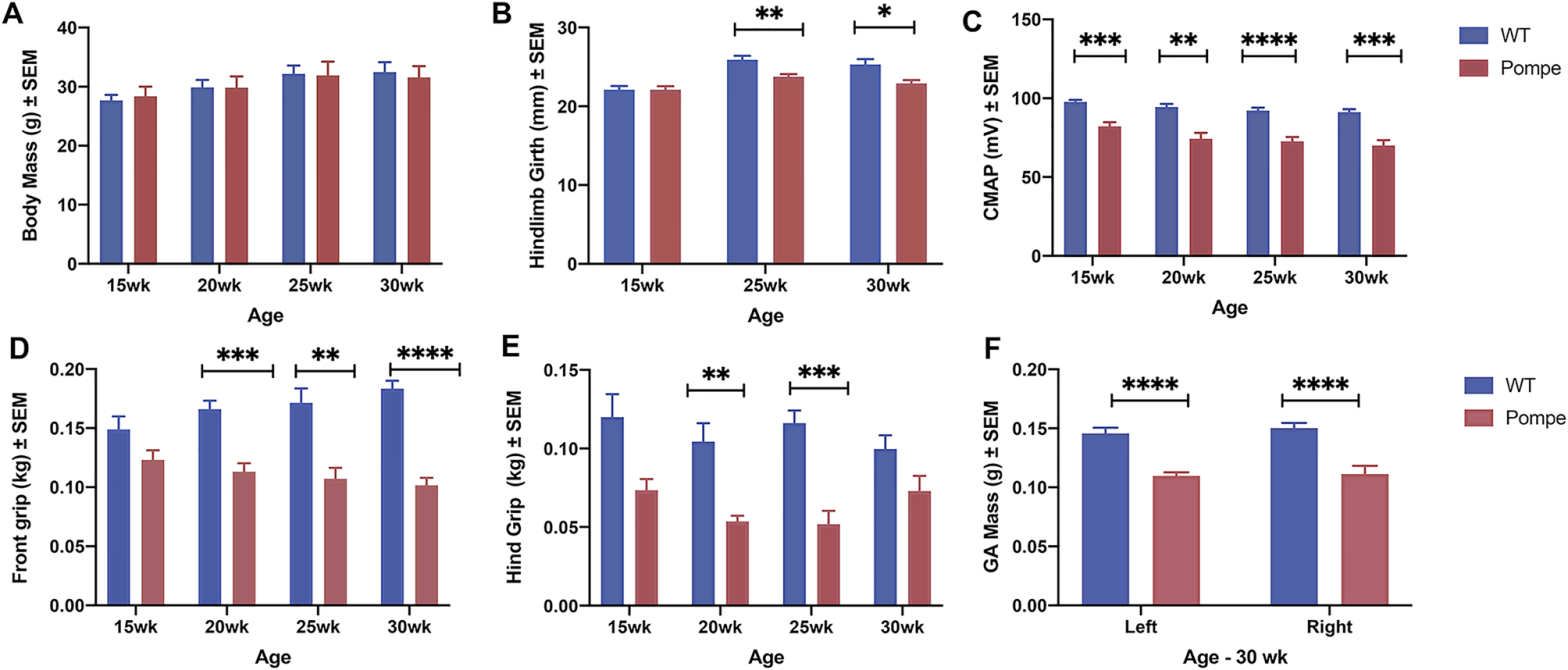
Physiological Data for WT and Pompe mice. Comparison of Body Mass (**A**), Hindlimb Girth (**B**), CMAP (**C**), Front Grip (**D**), and Hind Grip (**E**) between WT and Pompe mice at 15, 20, 25 and 30 weeks, and Gastrocnemius Wet Mass (**F**) at time of harvest (30 weeks). (N = 10 mice/group/time point). All data were reported as mean ± SEM. Statistical significance (Ordinary two–way ANOVA with Sidek’s Multiple comparisons test): * *p* < 0.05; ** *p* < 0.01; ***p < 0.001; ****p < 0.0001; ns not significant.

Figure 1 compares several basic physiological parameters in the Pompe and WT animals. Body mass did not change significantly in either group from 15 to 30 weeks of age (Fig. 1A), whereas hindlimb girth decreased in Pompe mice at both 25 and 30 weeks (Fig. 1B). In contrast, compound motor action potential (CMAP) was significantly lower in Pompe as compared to WT mice at all ages examined (Fig. 1C). Figure 1D and E provide the front and hind grip data and indicate a significant decrease in the strength of Pompe mice after 15 weeks. Figure 1F provides the wet mass of the GA muscle for Pompe and WT mice at the time of harvest (30 weeks of age), revealing the considerably lower muscle mass in the Pompe mice (average of 26% lower than WT). All means, standard errors of the mean (SEMs), and statistical comparisons for the physiological data can be found in Supplementary Table S1.

### Histological analysis and Myofiber size distribution

**Figure 2:**
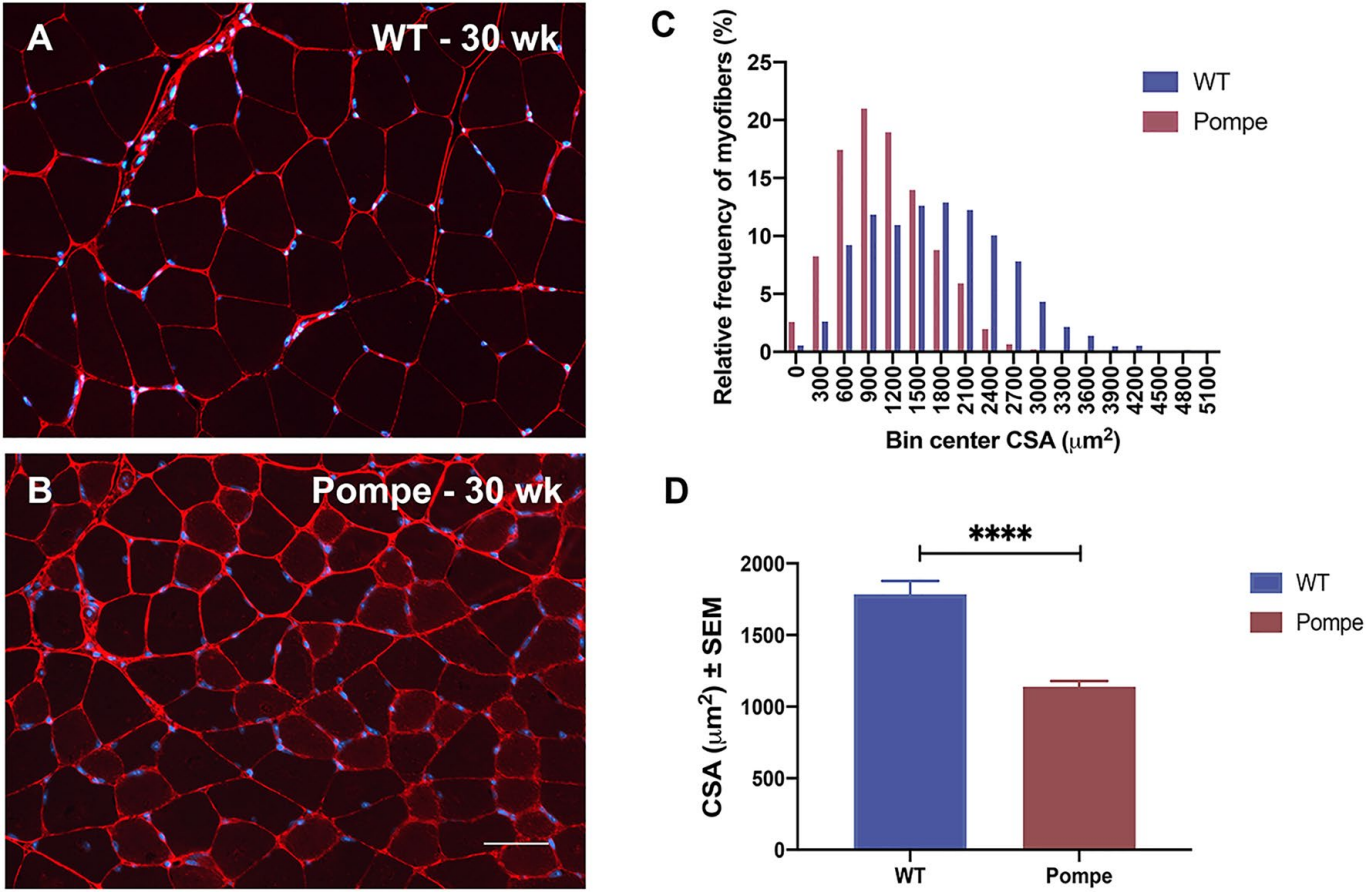
Histological analysis of myofiber CSA in WT and Pompe mice. Representative images of gastrocnemius (GA) muscle histology (stained with Collagen VI (red, cell membranes) and DAPI (blue, nuclei) for WT (**A**) and Pompe (**B**) mice at 30 weeks. The corresponding frequency distributions of the GA cross-sectional areas (CSA) from Pompe (N = 4097 fibers) and WT (N = 2678 fibers) are shown in the histogram (**C**). Note the major shift to smaller size fibers in the Pompe animals (Mean = 1125 ± 9) as compared to WT (Mean = 1740 ± 16) mice. (**D**) Mean GA muscle myofiber CSA in WT and Pompe mice at 30 weeks, reported as mean ± SEM. (Unpaired t-test). *p < 0.05; **p < 0.01; ***p < 0.001; ****p < 0.0001; ns not significant. Scale bar = 50 microns.

Figures 2A and 2B show representative histological images from Pompe and WT animals at 30 weeks of age revealing a marked difference in myofiber size between the WT and Pompe groups. Figure 2C provides interleaved histograms depicting the frequency distribution of the overall individual myofiber CSA data across both groups, quantifying the marked leftward shift in myofiber size distribution towards smaller fibers in the Pompe mice as compared to their WT counterparts. Figure 2D shows the mean GA muscle myofiber CSA for the Pompe and WT mice at 30 weeks. The mean myofiber CSA was 36% lower in the Pompe animals as compared to the WT (1784 ± 94 µm^2^ in WT mice to 1139 ± 41 µm^2^ in Pompe animals).

### Histological analysis and Glycogen Content in Gastrocnemius Muscle of Pompe Mice

**Figure 3:**
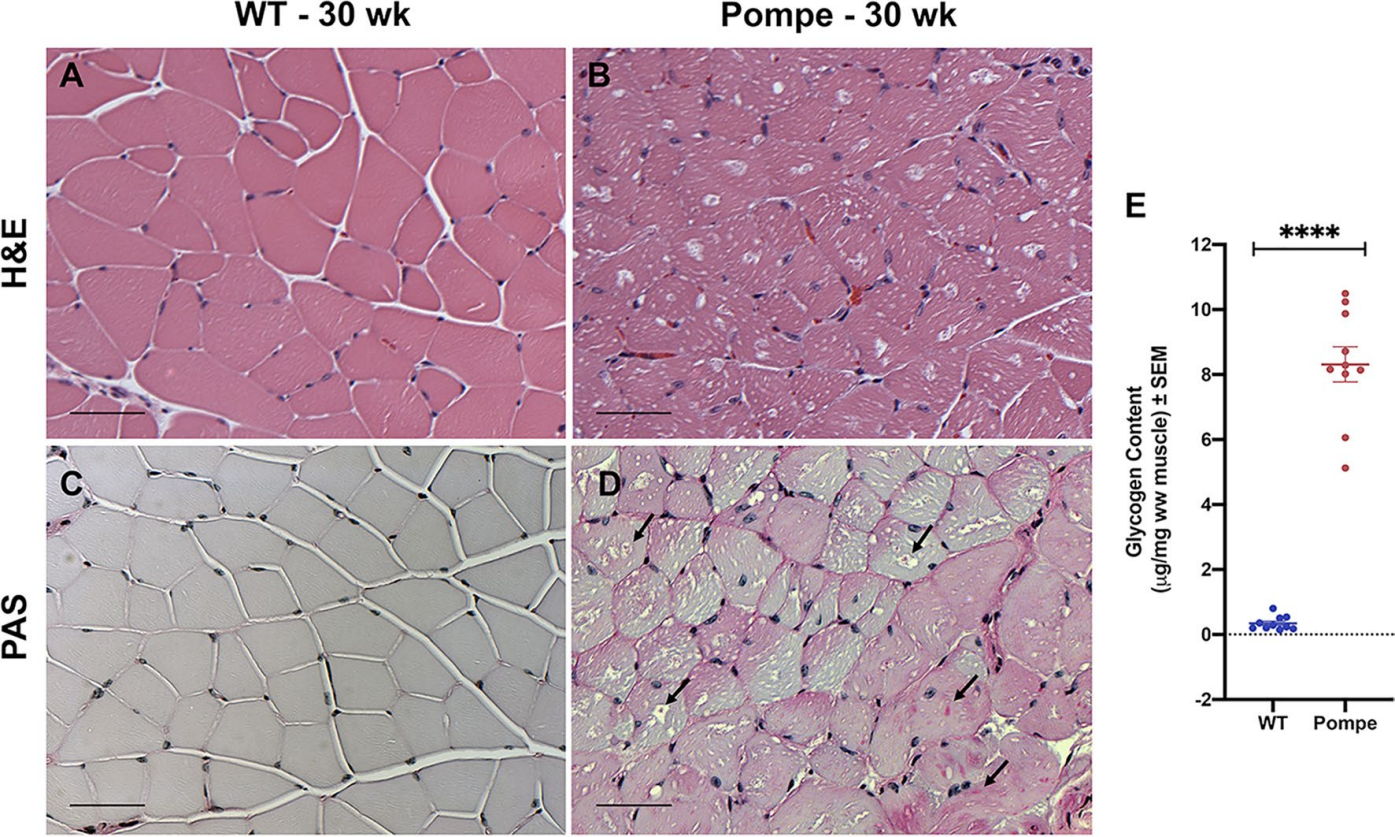
Histological analysis and biochemical determination of gastrocnemius muscle glycogen content in WT and Pompe mice. Representative examples of gastrocnemius (GA) muscle histology including standard H&E staining (**A**, **B**) as well as Periodic Acid Schiff (PAS) staining for glycogen for WT (**C**) and Pompe (**D**) mice at 30 weeks. (**E**) Glycogen Content in GA muscle from WT and Pompe mice as determined biochemically, reported as mean ± SEM with units µg glycogen/mg wet weight (ww) muscle. (Unpaired t-test). *p < 0.05; **p < 0.01; ***p < 0.001; ****p < 0.0001; ns not significant. Bar = 50 microns.

Figure 3 shows representative histological images from WT (Fig. 3A, C) and Pompe (Fig. 3B, D) animals, as well as the biochemical quantification of the GC in the GA muscles, from both groups (Fig. 3E). Standard H&E staining reveals scattered myofibers from Pompe muscle containing intra-cytoplasmic vacuoles (diameter ~ 10–20 microns) filled with basophilic amorphous material (Fig. 3B), not present in skeletal muscle from WT mice (Fig. 3A). In addition, tiny intra-cytoplasmic vacuoles can be seen in most Pompe muscle fibers (Fig. 3B). Figure 3C and D show representative results for PAS staining to identify glycogen deposition in the GA from WT (Fig. 3C) and Pompe (Fig. 3D) mice. In Fig. 3D, black arrows point to patches of PAS-positive material (red staining) indicating glycogen deposits within the Pompe muscle fibers as well as within numerous vacuoles located in Pompe skeletal muscle (Fig. 3D). No such glycogen deposits are visible in the WT muscle (Fig. 3C). The mean glycogen concentration increased from 0.345 ± 0.067 in the WT to 8.313 ± 0.542 (µg glycogen/mg wet weight (ww) muscle) in the Pompe mice, a 24-fold increase.

### Intrinsic electrical properties and correlation between conductivity and Glycogen content

**Figure 4:**
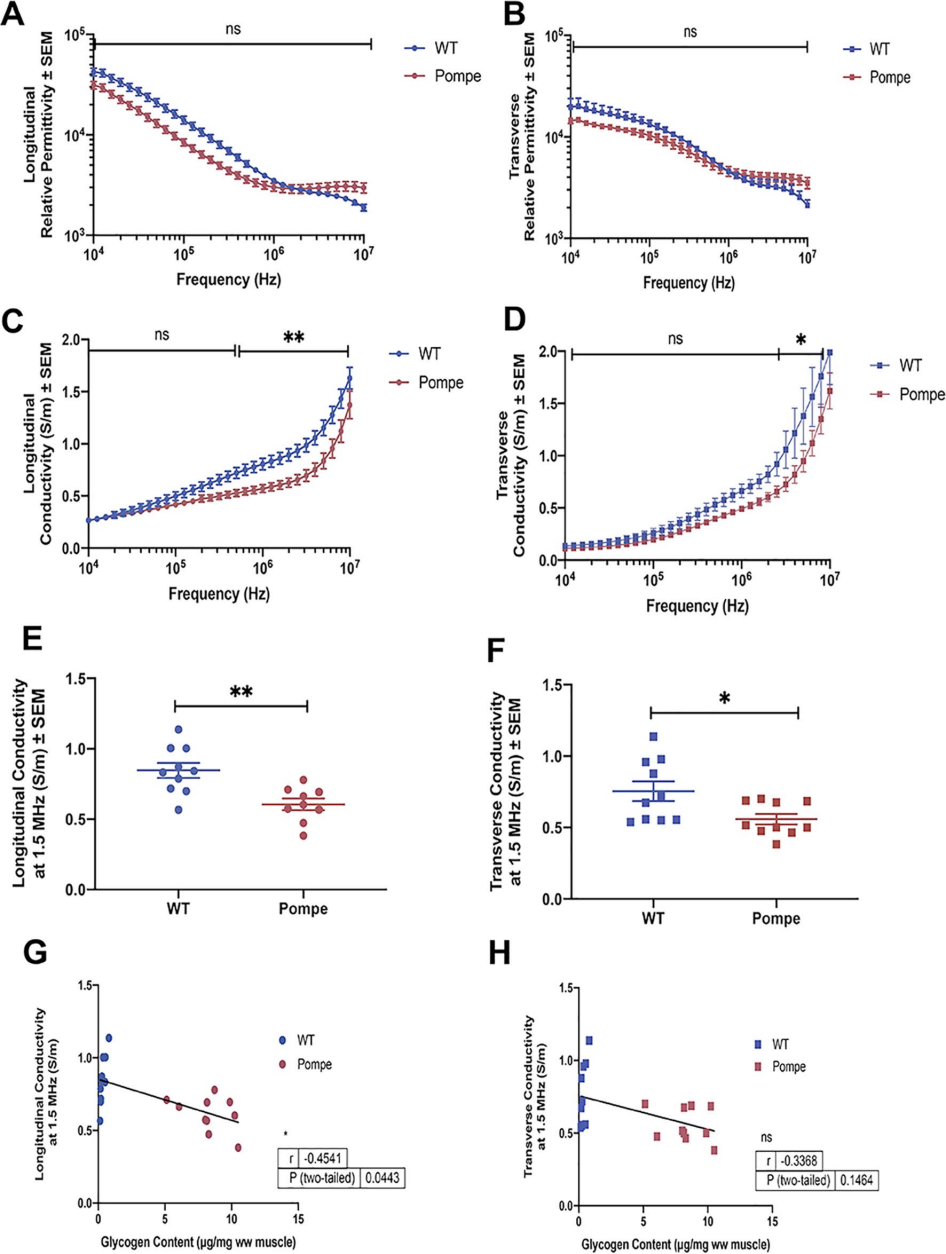
Dielectric properties of WT and Pompe gastrocnemius muscle. Relative permittivity values (mean ± SEM) from 10 kHz to 10 MHz in the longitudinal (**A**) and transverse (**B**) directions for WT and Pompe gastrocnemius (GA) muscle at 30 weeks showing no significant differences at any frequency. Conductivity values (mean ± SEM) from 10 kHz to 10 MHz in the longitudinal (**C**) and transverse (**D**) directions for the (GA) muscle from WT and Pompe mice at 30 weeks showing significant differences at frequencies > 1 MHz. GA muscle conductivity values at 1.5 MHz are depicted in the longitudinal (**E**) and transverse (**F**) directions for WT and Pompe mice at 30 weeks together with the respective mean ± SEM. Scatter plots showing the correlations between the glycogen content (GC) per animal and the measured conductivity at 1.5 MHz in both longitudinal (**G**) and transverse (**H**) directions. (Ordinary two–way ANOVA with Sidek’s Multiple comparisons test): *p < 0.05; **p < 0.01; ***p < 0.001; ****p < 0.0001; ns not significant.

The multifrequency relative permittivity and conductivity values, calculated using the calibrated ex vivo resistance and reactance data (provided in the Supplementary Data file), are shown in Fig. 4. Whereas no significant differences were detected in the relative permittivity values at any frequency in either direction (Fig. 4A and B), subtle yet significant reductions in both longitudinal (Fig. 4C) and transverse (Fig. 4D) conductivity can be seen in the Pompe mice compared to WT, particularly at frequencies > 1 MHz. The fact that this divergence is most prominent at high frequencies is consistent with the effect being mainly intracellular in nature. Longitudinal (Fig. 4E) and transverse (Fig. 4F) conductivity data at one specific frequency, i.e., 1.5 MHz, are shown for the individual Pompe and WT animals. Figure 4G and H provide the bivariate correlation analyses comparing the conductivity values at 1.5 MHz in the longitudinal (Fig. 4G) and transverse (Fig. 4H) directions with the measured GC in the GA muscle from each of these animals. A significant negative correlation between conductivity and GC was identified in the longitudinal orientation.

### Modelled parameters and the correlation with Glycogen content

**Figure 5:**
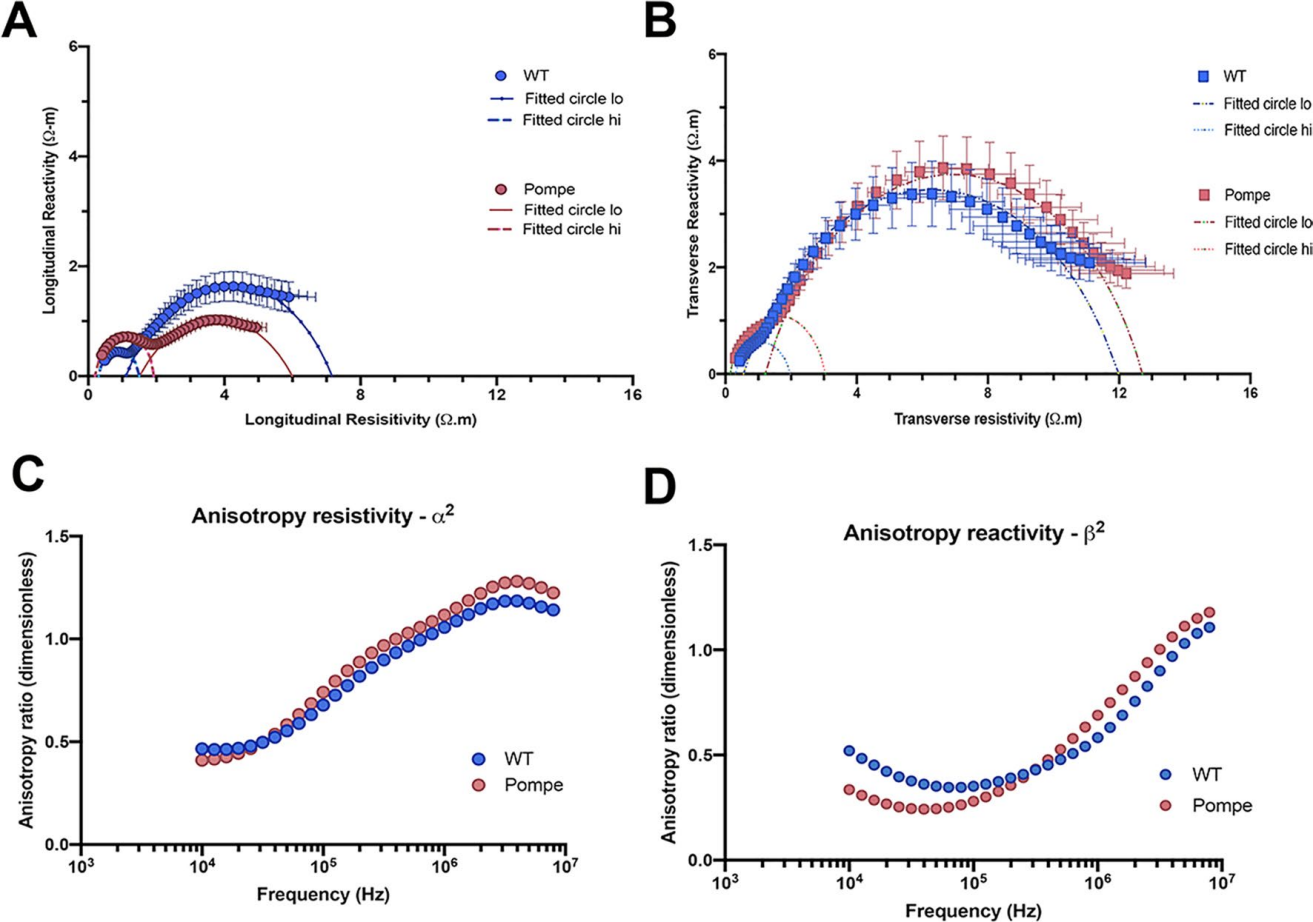
Resistivity and reactivity data for WT and Pompe gastrocnemius muscle fitted to the Cole–Cole resistivity model. Graphs showing the real (= Resistivity) versus the imaginary (= Reactivity) part of the complex resistivity spectrum for WT and Pompe GA muscle in the longitudinal (**A**) and transverse directions (**B**). Mean values and standard errors shown. The solid, dashed and dotted lines represent the model fit corresponding to respective high and low frequency arcs. Mean resistivity anisotropy ratio α^2^ (**C**) and mean reactivity anisotropy ratio β^2^ (**D**) for Pompe and WT mice indicating that the anisotropy ratios are unequal, i.e., α^2^ ≠ β^2^ at all frequencies.

The resistivity versus reactivity plots and their estimated fitted model parameters are shown in Fig. 5 and Table 1, respectively. Figure 5A and B show the real (resistivity, ρ) versus the imaginary (reactivity, τ) parts of the complex resistivity spectrum for Pompe and WT mice over the entire frequency range in both longitudinal and transverse directions, respectively. In the longitudinal direction, we were able to identify second high-frequency arcs in the complex resistivity data for both Pompe and WT mice. However, in the transverse direction, the high-frequency arcs are somewhat buried in the low-frequency arcs for both Pompe and WT animals. The solid, dashed, and dotted lines in Figs. 5A and B represent the model fit corresponding to respective high and low frequency arcs for the Pompe and WT animals. The values of the low and high frequency x-axis intercepts, corresponding to ρ_o_ and ρ_∞_, respectively, for the low (range 10 kHz–1 MHz) and high frequency (range 1–10 MHz) arcs are listed in Table 1, for both longitudinal and transverse directions. Individual plots of the resistivity anisotropy ratio, α^2^, and the reactivity anisotropy ratio, β^2^, versus frequency for Pompe and WT mice are shown in Fig. 5C and D, respectively, indicating that the anisotropy ratios are unequal, i.e., α^2^ ≠ β^2^, over the entire frequency range. Specifically, the muscle is more anisotropic at low frequencies and becomes more isotropic at higher frequencies when the anisotropy ratios get closer to 1. At high frequencies, it becomes anisotropic again, but now the direction of anisotropy (longitudinal vs transverse) changes.

**Table 1:** Estimated Cole–Cole Resistivity Model Parameters at low frequency (range from 10 kHz to 1 MHz) and high frequency (range from 1 to 10 MHz) including the extracellular and intracellular resistivity, in both longitudinal and transverse directions for the gastrocnemius muscle.

Direction	Cole–Cole Resistivity Model Parameters	WT—30 wk	Pompe – 30 wk	p value
Longitudinal	10 kHz to 1 MHz			
	ρ_0_ longitudinal (Ωm) < 1 MHz = *ρ*_extracellular_ longitudinal	7.162 ± 1.050	6.013 ± 0.518	0.3570 ns
	*ρ*_*∞*_ longitudinal (Ω m) < 1 MHz	1.109 ± 0.067	1.492 ± 0.115	0.0091 **
	*f*_*c*_ longitudinal (MHz) < 1 MHz	0.00774 ± 0.0011	0.00865 ± 0.0013	0.5947 ns
	α longitudinal (dimensionless) < 1 MHz	0.6464 ± 0.0134	0.5838 ± 0.035	0.1120 ns
	ρ_0—_*ρ*_*∞*_ longitudinal (Ω m) < 1 MHz	6.054 ± 1.060	4.521 ± 0.5295	0.2287 ns
Longitudinal	1 MHz to 10 MHz			
	ρ_0_ longitudinal (Ω m) > 1 MHz	1.500 ± 0.145	1.921 ± 0.117	0.0361 *
	*ρ*_*∞*_ longitudinal (Ω m) > 1 MHz	0.3047 ± 0.028	0.1989 ± 0.017	0.0044 **
	*f*_*c*_ longitudinal (MHz) > 1 MHz	2.642 ± 0.285	2.543 ± 0.397	0.8417 ns
	α longitudinal (dimensionless) > 1 MHz	0.8619 ± 0.028	0.8994 ± 0.021	0.2960 ns
	ρ_0—_*ρ*_*∞*_ longitudinal (Ω m) > 1 MHz	1.196 ± 0.166	1.722 ± 0.128	0.0215 *
	ρ _intracellular_ longitudinal (Ω m)	0.324 ± 0.032	0.202 ± 0.020	0.0061 **
Transverse	10 kHz to 1 MHz			
	ρ_0_ transverse (Ω m) < 1 MHz = *ρ*_extracellular_ transverse	11.99 ± 1.78	12.71 ± 1.44	0.7565 ns
	*ρ*_*∞*_ transverse (Ω m) < 1 MHz	0.5514 ± 0.131	1.212 ± 0.170	0.0065 **
	*f*_*c*_ transverse (MHz) < 1 MHz	0.0224 ± 0.0036	0.0234 ± 0.0026	0.8306 ns
	α transverse (dimensionless) < 1 MHz	0.6929 ± 0.032	0.7356 ± 0.030	0.3469 ns
	ρ_0—_*ρ*_*∞*_ transverse (Ω m) < 1 MHz	11.440 ± 1.796	11.50 ± 1.566	0.9806 ns
Transverse	1 MHz to 10 MHz			
	ρ_0_ transverse (Ω m) > 1 MHz	1.969 ± 0.237	2.639 ± 0.069	0.0193 *
	*ρ*_*∞*_ transverse (Ω m) > 1 MHz	0.3014 ± 0.022	0.1455 ± 0.029	0.0004 ***
	*f*_*c*_ transverse (Hz) > 1 MHz	1.34 ± 0.208	1.320 ± 0.397	0.9797 ns
	α transverse (dimensionless) > 1 MHz	0.783 ± 0.021	0.822 ± 0.042	0.4131 ns
	ρ_0—_*ρ*_*∞*_ transverse (Ω m) > 1 MHz	1.667 ± 0.244	2.476 ± 0.072	0.0076 **
	ρ _intracellular_ transverse (Ω m)	0.314 ± 0.026	0.128 ± 0.020	< 0.0001****

All values given as mean ± standard error of mean. Unpaired t test. *p < 0.05; **p < 0.01; ***p < 0.001; **** p < 0.0001; ns not significant.

Significant differences were found in several modelled resistivity parameters for Pompe versus WT mice, in both longitudinal and transverse directions, particularly at high frequency (range from 1 to 10 MHz) (Table 1) indicating a significant decrease in ρ_∞_ and a significant increase in both ρ_o_ and ρ_o_-ρ_∞_, in the Pompe versus the WT animals in both directions. This pattern is consistent with the presence of increasing poorly conductive intracellular material in the GA of the Pompe mice. Of note, no significant differences were observed for the model parameters *f*_*c*_ and α in either direction or in either frequency range.

**Figure 6:**
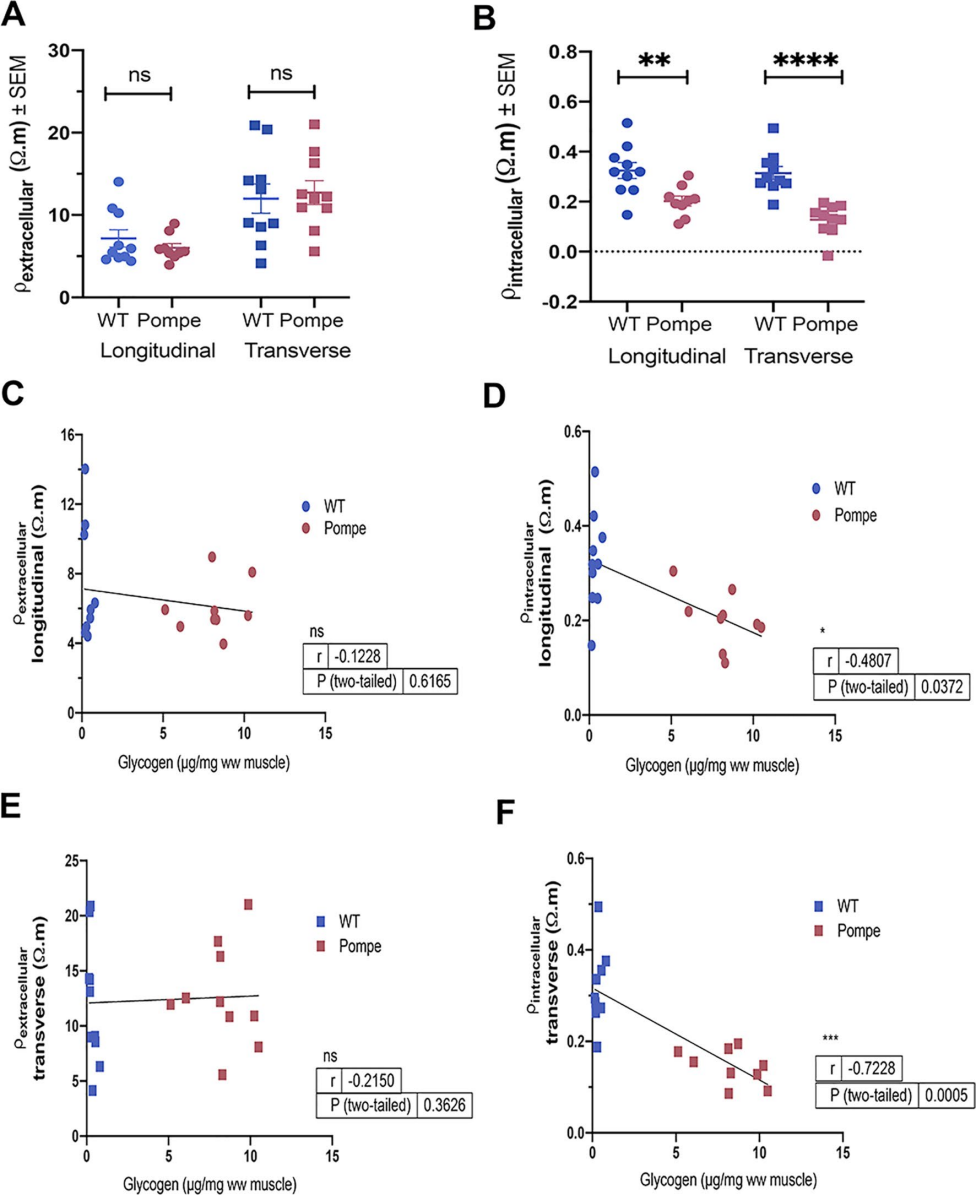
Extracellular and intracellular resistivity of WT and Pompe gastrocnemius muscle. Extracellular resistivity, ρ_extracellular_ (**A**) and intracellular resistivity, ρ_intracellular_ (**B**) values for WT and Pompe GA muscle at 30 weeks (shown with mean ± SEM). Scatter plots showing correlations between glycogen content (GC) and extracellular resistivity, ρ_extracellular_ (**C**, **E**) and intracellular resistivity, ρ_intracellular_ (**D**, **F**) in the longitudinal and transverse directions. (Unpaired t-test). * *p* < 0.05; ** *p* < 0.01; ***p < 0.001; **** *p* < 0.0001; ns not significant.

Values for the extracellular resistivity, ρ_extracellular_, for both WT and Pompe mice, in both the longitudinal and transverse directions are depicted in the scatter plot in Fig. 6A and in Table 1. No significant differences in ρ_extracellular_ between the WT and Pompe animals were detected in either direction. In contrast, significant differences in the intracellular resistivity, ρ_intracellular_, were detected between WT and Pompe mice in both directions, as depicted in Fig. 6B and Table 1. Figure 6C–F provide bivariate correlation analyses comparing longitudinal and transverse ρ_extracellular_ (Fig. 6C, E) and longitudinal and transverse ρ_intracellular_ (Fig. 6D, F) with the measured GC in the WT and Pompe animals. Significant negative correlations were identified between GC and ρ_intracellular_ in both longitudinal and transverse orientations.

## Discussion

The aim of the present work was to determine whether the electrical properties of skeletal muscle are different in a mouse model of glycogen storage disease (GSD) and assess the possible correlation between increased glycogen accumulation and any observed alterations in the electrical properties and/or modelled electrical impedance parameters. For this study we chose to use Pompe mice, the most widely utilized preclinical model of GSDII. Pompe mice, generated by insertion of a *neo* cassette within exon 6 of the *Gaa* gene^15^ exhibit features of both infantile and adult forms of GSDII including cardiomegaly, cardiomyopathy, skeletal muscle myopathy, and reduced survival (~ 50% of mortality at 10 months). Glycogen accumulation is typically observed at 3 weeks of age, and lysosomes increase in size and number thereafter. Disease progression, reported to occur slightly faster in the female versus male Pompe mice^16^, has been categorized by specific clinical signs^16^. Pompe disease progression can also be divided into distinct histological stages^17^: Stage 1—Glycogen-filled lysosomes located between intact myofibrils; Stage 2 – Enlarged glycogen-filled lysosomes; some leakage of glycogen into the cytoplasm; fragmentation of myofibrils; Stage 3 – Ruptured glycogen-filled lysosome membranes; definite glycogen in the cytoplasm; abnormal mitochondrial structure, few remaining myofibrils; Stage 4 – Contractile elements replaced by cytoplasmic glycogen; Stage 5 – Bloated cells, due to an influx of water, resulting in dissolution of glycogen. The female Pompe mice used in the present study were 30 weeks of age, and displayed no clinical manifestations of disease, i.e., had not yet reached clinical Grade 1. Nevertheless, both histological and biochemical data confirmed a significant increase in GC in the GA muscles of these animals, and, based on light microscopic analysis, the female Pompe mice used in this study can be categorized as histological Stage 1.

We hypothesized that intracellular myofiber abnormalities associated with early stage Pompe disease would produce alterations in the electrical properties of skeletal muscle. Using an ex vivo EIM approach, we were able to detect significant changes in the intrinsic volume electrical conduction properties of muscle at this relatively early stage in Pompe disease progression. Whereas both longitudinal and transverse relative permittivity remained unchanged at all frequencies, conductivity values in both directions showed a significant reduction in the Pompe animals, particularly at frequencies > 1 MHz. The data collected here can now be added to the database of permittivity properties of healthy and diseased skeletal muscle in murine models of SMA, DMD, diabetes, ALS and myofiber hypertrophy^18^.

Conductivity and relative permittivity data were then fitted to the complex resistivity version of the Cole–Cole model to provide the standard Cole–Cole resistivity parameters for the Pompe and WT mice. Differences in the longitudinal and transverse Cole–Cole resistivity parameters between Pompe and WT mice are apparent at both low (< 1 MHz) and high (> 1 MHz) frequencies. The base of the longitudinal low-frequency arc, defined by ρ_o_-ρ_∞_, is slightly, but not significantly, smaller in the Pompe mice as compared to WT; however, the base of the longitudinal high-frequency arc, ρ_o_-ρ_∞_, is significantly greater for the Pompe versus the WT mice. Similarly, in the transverse direction, the bases of the low-frequency arcs are not significantly different in the Pompe and WT animals, but once again, the base of the transverse high-frequency arc is significantly higher for the Pompe versus the WT mice. Finally, although no significant differences in ρ_extracellular_ were detected between the WT and Pompe animals, the significant decrease in the intracellular resistivity, ρ_intracellular_, detected in the Pompe mice, correlates with the increase in GC and might be attributed to an increase in intracellular water associated with the accumulating glycogen molecules^19–21^.

Additional anisotropic differences also merit discussion. Plots of the resistivity anisotropy ratio, α^2^, and the reactivity anisotropy ratio, β^2^, versus frequency indicate that the anisotropy ratios are unequal, i.e., α^2^ ≠ β^2^ for both Pompe and WT mice. The results also indicate that at low frequencies the muscle in both Pompe and WT mice is more anisotropic than at high frequencies, since both α^2^ and β^2^ increase towards 1 at high frequencies. These findings extend the set of anisotropy values calculated for the GA in several murine disease models^22^.

The present study has a number of limitations. First, only one age of Pompe mice was evaluated. It is possible that more severe pathological changes (e.g., increased glycogen accumulation, fragmentation of myofibrils), that have been reported to occur at later stages in disease progression^15^, would result in enhanced alterations in electrical properties and stronger correlation results, if muscles from older Pompe animals had been studied. Second, our total sample numbers were relatively small (N = 10 per group). Increasing the sample size would increase the statistical power of our analysis. Third, although unavoidable in these ex vivo measurements, since muscle tissue was measured shortly after death, post-mortem ischemia could have altered the electrical properties data as compared to living in situ muscle. Fourth, inconsistencies in accurately positioning the excised muscle in the dielectric cell inevitably causes some degree of inaccuracy in the directional dependence of the resultant conductivity and relative permittivity values. Fifth, we did not evaluate changes in mitochondrial content or the presence of increased autophagy in the Pompe mice that we studied at 30 weeks of age since these factors have been reported to occur in the Pompe mice only at much later times^23–25^. Nevertheless, such additional intracellular changes, if present, could also contribute to the observed alterations in the conductivity and the high frequency modelled parameters that we observed^9^. Finally, we acknowledge that our findings, especially the high frequency data, could be subject to a variety of errors (e.g., stray capacitances) inherent to the technique used^26^.

Despite these limitations, our ex vivo EIM study reveals differences in the electrical properties of Pompe muscle and their relationship to increased GC. Being able to evaluate alterations in the internal structures of the muscle cell would be valuable in a variety of pathological settings since such a strategy could serve as a tool to assess conditions affecting mainly sub-cellular muscle components such as lysosomes, the mitochondria, the t-tubule system or the sarcomeres themselves^27^. In addition, the technique could serve as a useful measure of drug efficacy in diseases attempting to restore normal intracellular architecture such as enzyme replacement therapy in Pompe disease^28^. Further investigations are thus warranted to evaluate if the ex vivo EIM findings presented here have translation using in vivo surface and needle EIM approaches for the assessment of this and other diseases exhibiting intracellular abnormalities including vacuolar and mitochondrial myopathies. Such information would provide additional support for the use of EIM as a non-invasive biomarker of muscle health. To this end, one very recent pilot study has correlated EIM outcomes with measures of muscle strength and function in several patients with late-onset Pompe disease^29^, suggesting that EIM could serve as a potential biomarker of clinical change and response to interventions in this disease.

## Methods

### Animals

All experimental procedures were approved by the Institutional Animal Care and Use Committee at Beth Israel Deaconess Medical Center (Protocol Number 031–2019) and performed in accordance with guidelines set forth in The *Guide for the Care and Use of Laboratory Animal, 8th edition, 2011* of the National Institutes of Health. All authors complied with the ARRIVE guidelines 2.0. Female mice homozygous (N = 10) or wild type (N = 10) for Gaa^tm1Rabn^ (B6;129-*Gaa*^*tm1Rabn*^/J, also known as Pompe^*6neo*/*6neo*^ (Pompe)); Strain #004154) were obtained from Jackson Labs (Bar Harbor ME) and aged to 30 weeks in order to ensure elevated glycogen content, which occurs naturally as these homozygous animals age. All animals were fed standard chow ad libitum.

### Grip strength and CMAP amplitude

Forelimb and hindlimb grip strength^7^ and Compound Muscle Action Potential (CMAP) amplitudes^30^ were each measured as previously described.

### Gastrocnemius muscle extraction

Mice were euthanized by CO_2_ from gas cylinder. After excision of the entire GA muscle, wet mass was determined using a standard analytical balance and height was recorded with a micrometer. GA was cut to approximately 5 × 5  mm^2^ (with variable height) to fit into the dielectric cell used for ex vivo impedance measurements, described below.

### EIM methods

All electrical impedance measurements were made with the mView impedance spectroscopy system (Myolex Inc., Boston, MA) sweeping the frequency of the electrical current applied. In total, 41 logarithmically spaced frequencies were measured from 1 kHz to 10 MHz. Data was collected via an ex vivo approach using a Plexiglass dielectric measuring cell^8^. The excised GA was first placed in the cell with the fibers oriented perpendicularly to the metal plates (for longitudinal myofiber measurements), then removed and placed with the fibers parallel to the plates (for transverse myofiber measurements).

### Muscle histology

Following ex vivo impedance measurements, left GA muscles were placed in 10% buffered formalin and fixed for at least 48 h. Samples were then embedded in paraffin blocks, sectioned into 10-μm slices, and stained with either Hematoxylin & Eosin (H&E) or Periodic Acid-Schiff (PAS) by standard methods and imaged at 20X and 40X by light microscopy. Additional paraffin sections were stained with anti–collagen VI antibody (Abcam ab6588) to identify the myocyte cell membranes and 4',6-diamidino-2-phenylindole (DAPI) to detect nuclei. The myofiber CSA was determined as previously described^7^. Sections were imaged at 20X with an epifluorescence microscope (AxioImager M1, Zeiss) and myofiber area was measured using muscle morphometry plug-in (developed by A. Sinadinos using Eclipse IDE) in FIJI, the open source image processing software (ImageJ version 2.0.0-rc-68/1.52d; W.S. Rasband, ImageJ, National Institutes of Health, Bethesda, Maryland [https://imagej.nih.gov/ij/], 1997–2016). On average, 249 myofibers per WT muscle) and 406 myofibers (per Pompe muscle) were counted per animal, for total number of 2678 myofibers for WT mice and 4097 myofibers for Pompe mice at 30 weeks.

### Glycogen assay

Following ex vivo impedance measurements, the entire right GA muscles were immediately snap frozen and a small portion of the muscle (~ 10–15 mg) was later analyzed for GC, using the Glycogen Assay Kit II Colorimetric Kit (#Ab169558, Abcam, Cambridge, MA) and a microplate reader (Fisherbrand accuSkan GO UV/Vis Microplate Spectrophotometer, Fisher Scientific, Pittsburgh, PA) according to the manufacturer’s instructions including a background control. Results are reported as µg glycogen/mg wet weight (ww) muscle.

### Data analysis, calibration, and parameterization

Resistance (R) and reactance (X) values were collected across the entire frequency range. Prior to the formal analysis, we removed any spurious EIM data (i.e., multifrequency curves exhibiting negative values over a portion of the frequency range or highly aberrant shapes) yielding a sample size of N = 10 (WT) and N = 9 (Pompe). Next, we calibrated the impedance measuring setup to compensate for potential high frequency measurement errors due to stray capacitances affecting the wires connecting the device to the electrodes. For this, impedance measurements were made with normal saline solution placed into the same dielectric cell. From the calibrated impedance values, the constituent volume conduction properties of muscle, i.e., the conductivity (σ), in Siemens/m, and the relative permittivity (ε_r_), a dimensionless value were calculated for each individual frequency of applied current, as previously described^18^.

Obtained conductivity and relative permittivity data were converted into their equivalent resistivity, ρ, in Ω.m, and reactivity, τ , in Ω.m, values^9^, and their directional dependence – a concept known as electrical anisotropy, was calculated^22^. At each frequency, the longitudinal resistivity was divided by the transverse resistivity (ρ_L_/ρ_T_) to determine the resistivity anisotropy ratio, α^2^. Similarly, at each frequency the longitudinal reactivity was divided by the transverse reactivity (τ_L_ /τ_T_) to determine the reactivity anisotropy ratio, β^2^.

Longitudinal and transverse resistivity and reactivity data were then fitted to the Cole–Cole resistivity model^31^ using MATLAB (Mathworks, Natick, MA, USA) as described^9^, with a weighted nonlinear least squares method^32^ to provide the standard Cole–Cole parameters, including resistivity at 0 Hz and infinite frequency, ρ_o_ and ρ_∞_ respectively, (both in Ω.m); center frequency *f*_*c*_ (in MHz), and α (dimensionless). These parameters are associated with cell density (ρ_o_ and ρ_∞_), myocyte size (*f*_*c*_) and cell size distribution (α).

Finally, ρ_o_, the resistivity at 0 Hz, assumed to be equivalent to the resistivity of the extracellular fluid, ρ_extracellular_, and ρ_∞_, the resistivity at infinite frequency, assumed to be the resistivity of the whole volume when current is flowing through both intracellular and extracellular compartments, were used to calculate the longitudinal and transverse resistivity of the intracellular compartment, ρ_intracellular_, (in Ω.m), based on Eq. (1)^3^:

1 $$1/\uprho \,extracellular+1/\uprho\, intracellular=1/\uprho \infty$$

### Statistical analyses

Basic statistical analyses of the physiological, histological, biochemical, impedance values, the conductivity and relative permittivity data, and the Cole parameters were performed using GraphPad Prism V8 (GraphPad Software, Inc. La Jolla, CA) using the most appropriate tests (unpaired t-tests or one-way ANOVA followed by post hoc tests, as described). Unless otherwise noted, all data were reported as mean ± SEM. Multiple group comparisons were performed by one-way ANOVA with Tukey’s multiple comparisons test. Multifrequency EIM values were compared using the two-way ANOVA followed by Sidak’s multiple comparison test. For correlation analyses, the Spearman correlation coefficient, r, was calculated. Statistical significance was based on *p* < 0.05. Asterisks in figures, indicate statistical significance * p < 0.05, ** p < 0.01, *** p < 0.001, **** p < 0.0001.

## Supplementary Information

Supplementary Information 1.

Supplementary Information 2.

## Data Availability

All data generated or analyzed during this study are included in the Supplementary Data file.
