# Supplementary material for: Altered electrical properties in skeletal muscle of mice with glycogen storage disease type II
Source: Sci Rep. 2022 Mar 29;12:5327. doi: 10.1038/s41598-022-09328-0 (PMC8964715; doi:10.1038/s41598-022-09328-0)
Supplement: Supplementary file 2 — Supplementary Information 2. [file 41598_2022_9328_MOESM2_ESM.docx]

| **Physiological and Electrical properties** | **WT – 30 weeks** | **Pompe – 30 weeks** | **p value** |
| --- | --- | --- | --- |
| Body mass (g) | 32.486±1.679 | 31.582±1.902 | 0.9943 ns |
| CMAP (mV) | 91.240±1.781 | 70.020±3.414 | 0.0003 *** |
| Girth (mm) | 25.307±0.671 | 22.916±0.409 | 0.0246 * |
| Front Grip (kg) | 0.184±0.007 | 0.102±0.006 | <0.0001 **** |
| Rear Grip (kg) | 0.100±0.009 | 0.073±0.010 | 0.1995 ns |
| Gastrocnemius wet mass (g) | 0.148±0.0045 | 0.111±0.005 | <0.0001 **** |
| Normalized GA mass (g/g) | 0.0047±0.00002 | 0.0037±0.0003 | 0.0068 ** |
| Mean Myofiber CSA (µm^2^) | 1784±94 | 1139±41 | <0.0001 **** |
| Glycogen Content (μg glycogen/mg ww muscle) | 0.345±0.067 | 8.313±0.542 | <0.0001 **** |
| Longitudinal Conductivity at 1.5 MHz (S/m) | 0.861±0.057 | 0.605±0.041 | 0.0023 ** |
| Longitudinal Relative Permittivity at 1.5 MHz (dimensionless) | 2975±123 | 2909±279 | 0.8326 ns |
| Transverse Conductivity at 1.5 MHz (S/m) | 0.741±0.076 | 0.559±0.037 | 0.0391 * |
| Transverse Relative Permittivity at 1.5 MHz (dimensionless) | 3749±344 | 4195±448 | 0.4406 ns |

**Supplementary Table S1. Summary of Physiological Data and Electrical Properties of WT and Pompe mice. All values given as mean ± standard error of mean. Unpaired t test.** * p<0.05; ** p<0.01; ***p< 0.001; **** p<0.0001; ns not significant.
